# Arbuscular Mycorrhizal Fungal Hyphae Alter Soil Bacterial Community and Enhance Polychlorinated Biphenyls Dissipation

**DOI:** 10.3389/fmicb.2016.00939

**Published:** 2016-06-15

**Authors:** Hua Qin, Philip C. Brookes, Jianming Xu

**Affiliations:** ^1^Institute of Soil and Water Resources and Environmental Science, Zhejiang Provincial Key Laboratory of Subtropical Soil and Plant Nutrition, Zhejiang UniversityHangzhou, China; ^2^Key Laboratory of Soil Contamination Bioremediation of Zhejiang Province, School of Environmental and Resource Sciences, Zhejiang A & F UniversityLin’an, China

**Keywords:** *Glomus mosseae*, microbial community, mycorrhizal hyphae, polychlorinated biphenyls, *bph* gene

## Abstract

We investigated the role of arbuscular mycorrhizal fungal (AMF) hyphae in alternation of soil microbial community and Aroclor 1242 dissipation. A two-compartment rhizobox system with double nylon meshes in the central was employed to exclude the influence of *Cucurbita pepo* L. root exudates on hyphal compartment soil. To assess the quantitative effect of AMF hyphae on soil microbial community, we separated the hyphal compartment soil into four horizontal layers from the central mesh to outer wall (e.g., L1–L4). Soil total PCBs dissipation rates ranged from 35.67% of L4 layer to 57.39% of L1 layer in AMF inoculated treatment, which were significant higher than the 17.31% of the control (*P* < 0.05). The dissipation rates of tri-, tetrachlorinated biphenyls as well as the total PCBs were significantly correlated with soil hyphal length (*P* < 0.01). Real-time quantitative PCR results indicated that the *Rhodococcus*-like *bphC* gene was 2–3 orders of magnitude more than that of *Pseudomonas*-like *bphC* gene, and was found responded positively to AMF. Phylogenetic analyses of the 16S rDNA sequenced by the Illumina Miseq sequencing platform indicated that AMF hyphae altered bacterial community compositions. The phylum *Betaproteobacteria* and *Actinobacteria* were dominated in the soil, while *Burkholderiales* and *Actinomycetales* were dominated at the order level. Taxa from the *Comamonadaceae* responded positively to AMF and trichlorinated biphenyl dissipation, while taxa from the *Oxalobacteraceae* and *Streptomycetaceae* responded negatively to AMF and PCB congener dissipation. Our results suggested that the AMF hyphal exudates as well as the hyphae *per se* did have quantitative effects on shaping soil microbial community, and could modify the PCBs dissipation processes consequently.

## Introduction

Arbuscular mycorrhizal fungi (AMF) are ubiquitous in the terrestrial ecosystem. It is estimated that 250,000 species of plants worldwide, including many arable crops, are capable of forming the symbiosis with this group of fungi (Smith and Read, 2008). AMF receiving carbon from their host, and in return, delivering nutrients and water back. It has been estimated that in natural ecosystems plants colonized with AMF may invest 10–20% of the photosynthetically fixed carbon in their fungal partners (Johnson et al., 2002), and this significant input of energy and carbon into the soil ecosystem could be crucial to microorganisms associated with the AMF.

As the extraradical hyphae of AMF provides a direct pathway for translocation of photosynthetically derived carbon to the soil, the continuous provision of energy-rich compounds, coupled with the large surface area of the hyphae that intact with the surrounding soil environment (hyphosphere) provide important niches for bacterial colonization and growth (Toljander et al., 2007). The AMF hyphae may have both positive (Johansson et al., 2004; Toljander et al., 2007) and negative (Welc et al., 2010) effects on microbial growth. Using quantitative real-time PCR method, we also detected significant higher 16S rDNA abundance in both the bulk and the rhizosphere soils of *Acaulospora laevis* and *Glomus mosseae* inoculated treatments (Qin et al., 2014a). Compared to the quantitative changes in bacterial numbers, more studies have demonstrated shifts in bacterial community occurred in the presence of AMF (Lioussanne et al., 2010; Leigh et al., 2011; Nottingham et al., 2013; Nuccio et al., 2013). However, some studies also found AMF have no discernable effect on the composition of the microbial community present in litter-containing soil (Hodge et al., 2001; Herman et al., 2012). Though some bacterial species can utilize the hyphae themselves as substrate (Toljander et al., 2007), it is trusted that the changes in the bacterial community in the hyphosphere were not due to the amount of mycelium *per se*, suggesting that the qualitative effects (e.g., composition of exudates) of the AMF on the hyphosphere bacteria are more important than the quantitative development of AMF hyphae (Andrade et al., 1997; Johansson et al., 2004). Carbohydrates (Hooker et al., 2007; Toljander et al., 2007) and citric acid (Tawaraya et al., 2006) were detected in mycorrhizal hyphal exudates. In such a manner, the change of soil microbial biomass and the modification of the soil microbial community could be mainly dependent on quantitative and qualitative changes of hyphal exudates (Filion et al., 1999; Toljander et al., 2007; Lioussanne et al., 2010).

Few studies have explicitly studied how AMF influence the soil bacterial community responsible for PCB degradation. AMF have been proved having great potential on the rhizoremediation of organic pollutants through mycorrhizosphere (the zone influenced by both the root and the mycorrhizal fungus) effect (Joner and Leyval, 2003). Teng et al. (2010) reported that *Glomus caledonium* and *Rhizobium meliloti* had a significant synergistic effect on field soil PCBs removal when compared with non-inoculated alfalfa treatment. In our previous study, the dissipation rates of Aroclor 1242, both in bulk and rhizosphere soil, were greatly enhanced by the inoculation of *Acaulospora laevis* or *Glomus mosseae* (Qin et al., 2014a). The results also demonstrated a significant contribution of *Actinobacteria* to the PCB congener profiles in the bulk soil, indicating the important role of mycorrhizal extraradical hyphae on shaping bacterial community and PCB congener profile compositions. However, we did not separate the mycorrhizosphere and hyphosphere effects in the study. To our knowledge, no previous studies have investigated the effect of AMF on the hyphosphere soil microbial community mediating PCBs dissipation. The AMF hyphal exudates not only contain low-molecular-weight sugars and organic acids, but also unidentified high-molecular-weight polymeric compounds (Toljander et al., 2007). These compounds are energy-rich, and can stimulate or otherwise affect the growth of hyphosphere soil bacteria (Toljander et al., 2007). There are also the possibilities that some of the exudates have the similar chemical structure to PCBs, and may act as inducers for PCBs degradation (Singer et al., 2003).

We investigated how the AM fungus, *Glomus mosseae*, altered the bacterial communities and PCB congeners dissipation in soil spiked with Aroclor 1242. To accomplish this, we used a two-chamber rhizobox to allow AMF hyphae access to the Aroclor 1242 contaminated soil (Supplementary Figure S1). Furthermore, we separated the contaminated soil into four horizontal layers from the central mesh to the outer wall to assess the quantitative effect of mycorrhizal hyphae on soil bacterial community and PCBs dissipation. We hypothesized that different quantity of AMF hyphae results different native bacterial community and PCB congener profile compositions. The results may help us clarify the importance of AM hyphae on soil PCBs dissipation, and promote the field application of AM fungi on soil PCBs bioremediation.

## Materials and Methods

### Experiment Design

The soil classified as Fe-accumuli-Anthrosol was collected from a paddy field in Zhejiang Province, China. Soil pH (soil to water ratio, 1:2.5), organic carbon, and total N were 7.14, 14.3, 0.72 g kg^-1^, respectively. There was no PCB congeners detected in the soil. The air-dried soil was mixed thoroughly with 15 mg kg^-1^ Aroclor 1242 dissolved in hexane and placed in an exhaust hood for 48 h with periodic mixing to allow the hexane to evaporate.

The experiment was carried out in an artificially controlled climate chamber, with a day/night (12/12 h) temperature 30/22°C (10,000 Lux), and a relative humidity 50%. The experimental plants of zucchini (*Cucurbita pepo* L. cv black beauty) were grown in polyvinyl chloride (PVC) rhizoboxes (15 cm × 10.5 cm × 15 cm) divided by two nylon meshes (<35 μm) into one root compartment (15 cm × 5 cm × 15 cm) and one hyphal compartment (15 cm × 5 cm × 15 cm). The two nylon meshes were placed in the central of the rhizobox, with 0.5 cm space between them to prevent the transfer of root exudates to the hyphal compartment soil (Supplementary Figure S1). Seeds of the host plant were surface sterilized in a 1:10 solution of sodium hypochlorite (5% available chlorine) for 15 min and rinsed three times with deionised water. Seedlings were pre-cultured in sterilized sand in a greenhouse. Plantlets were then transplanted into the root compartments of rhizoboxes containing sterilized sand after 1 week. The hyphal compartments of the rhizoboxes were then filled with 1.5 kg of Aroclor 1242 spiked soil. Soil moisture content was adjusted to 50% of water-holding capacity (WHC) before use.

Arbuscular mycorrhizal inoculum used in this study was *Glomus mosseae* M47V which obtained from the International Culture Collection of (Vesicular) Arbuscular Mycorrhizal Fungi, France. The inoculum was propagated on sudangrass [*Sorghum sudanese* (Piper) Stapf.] grown in an autoclaved (121°C for 1 h on three successive days) sandy soil for 3 months. At the same time, the control non-mycorrhizal inoculum was also prepared with the same sterilized substratum on which sudangrass was cultivated. The root compartment of each AMF-inoculated treatment or non-mycorrhizal control was mixed with 150 g of mycorrhizal or non-mycorrhizal inoculums before transplanting. There were three replicates with both AMF inoculated treatments and non-mycorrhizal control. The rhizoboxes were arranged in a completely randomized design. During the entire period of the experiment, the soil moisture was maintained at about 50% of the field WHC and the sands in root compartments were fertilized with 30 ml of 10% Hoagland’s solution weekly.

At day 40 after transplanting, the plants were harvested, with shoots and roots sampled separately to analyze root colonization rate and plant biomass. Soils in each hyphal compartment of AMF inoculated treatment were separated into four horizontal layers (1.25 cm thick) by knife, for example, L1–L4 layers were separated according to the distance from the central nylon mesh to the boundary of the rhizobox. The non-mycorrhizal control soil was not separated into different layers. All soil samples were homogenized separately before analyses. Soil samples used for PCB residue and phospholipid fatty acid (PLFA) determination and DNA extraction were freeze-dried, and stored at -40°C. Subsamples for external mycelium determination were stored at 4°C.

### AMF Growth Analyses

Root mycorrhizal colonization rate was estimated after clearing and staining, using the acid fuchsin staining-grid intersect method (Giovannetti and Mosse, 1980). The external mycelium of AM fungi was extracted from the soil samples and measured by the filtration-gridline method described by Schreiner et al. (1997). Soil AMF biomass was evaluated using the PLFA biomarker 16:1ω5c (Olsson et al., 1999). PLFA was performed using a modified technique (Wu et al., 2009) similar to that of Frostegård and Bååth (1996). Brifely, fatty acids were extracted from 3 g freeze-dried soil with a chloroform:methanol:citrate buffer. Fatty acids were then separated on a solid phase extraction column (Supelco, Inc., Bellefonte, PA, USA). Following methylation of the phospholipids, the PLFA methyl esters were separated and identified by gas chromatography (N6890, Agilent, USA) combined with a MIDI Sherlocks microbial identification system (Version 4.5, MIDI, USA). The fatty acid 19:0 phosphatidylcholine was added as an internal standard before methylation. Fatty acid peak areas were converted to nmol g^-1^ soil using internal standards.

### PCBs Extraction and Analyses

Soil PCBs were extracted and analyzed using ultrasonic solvent extraction method described by Qin et al. (2014b). All procedures were carried out under room temperature. Briefly, 3 g freeze-dried soil was grounded and spiked with the surrogate standard [Decachlorobiphenyl (DCPB)] for PCB analysis. Then the soil samples were extracted three times by ultrasonics for 30 min (100 KHz, 25°C) in 30 ml 1:1 (v/v) acetone and hexane. The combined extracts were then further purified on a Florisil column with 40 ml hexane, and condensed to 1 ml under a gentle N_2_ stream. Soil extracts were analyzed for total PCBs using GC/mass spectrometry (MS; N6890/5975B, Agilent, USA), which was equipped with a HP-5MS capillary column (30 m length, 250 μm i.d., 0.25 μm film thickness). All measurements were carried out at full-scan mode with a mass range *m*/*z* from 50 to 450. The initial oven temperature was 80°C for 0.8 min, then increased at 25°C min^-1^ to 140°C for 1 min, followed by 10°C min^-1^ until the final temperature of 260°C, held for 5 min. Helium was used as the carrier gas at a flow rate of 1.0 ml min^-1^. A representative GC–MS chromatograph for PCB congeners profile is shown in Supplementary Figure S2.

### qPCR Assay and High Throughput Sequencing

Total DNA was extracted from 0.5 g of freeze-dried soil using a MoBio Powersoil^TM^ soil DNA isolation kit (MoBio Laboratories, Carlsbad, CA, USA) according to the manufacturer’s protocol. The abundance of the bacteria and PCB-degrading community was estimated from real-time qPCR assays targeting 16S and *bph* genes in soil DNA extracts. The qPCR and standard curve determination were act as described by our previous study (Qin et al., 2014a).

Soil microbial community was identified by amplifying approximately 400 bp of the 16S rDNA genes using barcoded primers 519F and 907R (Biddle et al., 2008) and sequencing on the Illumina Miseq platform. The sequences were processed and analyzed using the Quantitative Insights Into Microbial Ecology (QIIME) 1.4.0-dev pipeline^1^ (Caporaso et al., 2010) as we described previously (Qin et al., 2014a). Taxonomy was assigned to bacterial OTUs against a subset of the Silva 104 database^2^. OTU representative sequences were classified using RDP’s classifier with a 50% bootstrap confidence (Wang et al., 2007).

### Statistical Analyses

All PCB concentrations and gene abundances are reported on a dry weight basis and are the means of 3 replicate analyses. The data were subjected to one-way ANOVA using SPSS 18.0 software. The results were expressed as mean values with standard deviations, and compared by the Duncan’s multiple range test. Statistical significance was determined at the 95% level (*P* < 0.05). Pearson’s correlation coefficients (*r*) were calculated to assess the relationships of PCB congeners with soil AMF biomass and microbial taxonomy distributions. Principal component analysis (PCA) of soil PCB congener profiles and bacterial community compositions were performed using Canoco for Windows 4.5 (Ter Braak and Šmilauer, 2002).

## Results and Discussion

### AMF Biomass in the Rhizoboxes

Zucchini root mycorrhizal colonization rate in the *Glomus mosseae* M47V inoculated treatment was 52% ± 6% after 40 days of growth, while no mycorrhizal colonized roots were detected in the non-mycorrhizal control, indicating the zucchini could be a good host plant for *Glomus mosseae* M47V colonization. Soil mycorrhizal hyphal lengths were significant higher (*P* < 0.05) in the L1-L3 layers of the AM-inoculated treatment when compared to the L4 layer, and no mycorrhizal hypha was detected in the control (**Table 1**). Moreover, the hyphal length in L3 layer was lower than that in the L1 and L2 layers, though there were no significant differences. The result proved our hypothesis that the mycorrhizal hyphae can penetrate the double meshes and the hyphal length decreasing with the increasing distance from the meshes. Similar decreasing trend was also found when using PLFA biomarker 16:1ω5c as an indicator of AMF biomass (**Table 1**). However, AMF PLFA biomarker was also detected in the non-mycorrhizal control. One possible reason is the AMF spores which still existed because the soil we used was not sterilized. Furthermore, the biomarker 16:1ω5c can also be detected in some gram-negative bacteria according to Zelles (1997), which could lead to the overestimation of soil AMF biomass.

**Table 1:** Soil arbuscular mycorrhizal fungal hyphal length and phospholipid fatty acid (PLFA) biomass in different soil layers under mycorrhiza inoculation treatments and the control (mean ± SD; *n* = 3).

	Hyphal length	PLFA biomass
Control	ND	1.09 ± 0.09 ab
AM-L1	3.27 ± 0.40 a	1.57 ± 0.28 c
AM-L2	3.24 ± 0.26 a	1.26 ± 0.15 bc
AM-L3	2.71 ± 0.23 a	0.91 ± 0.10 ab
AM-L4	1.77 ± 0.47 b	0.81 ± 0.22 a

*Different *lowercase letters* in the same column indicate significant differences between the control and different soil layers by Duncan’s multiple range test at *P* < 0.05*.

### Soil PCBs Dissipation

**FIGURE 1:**
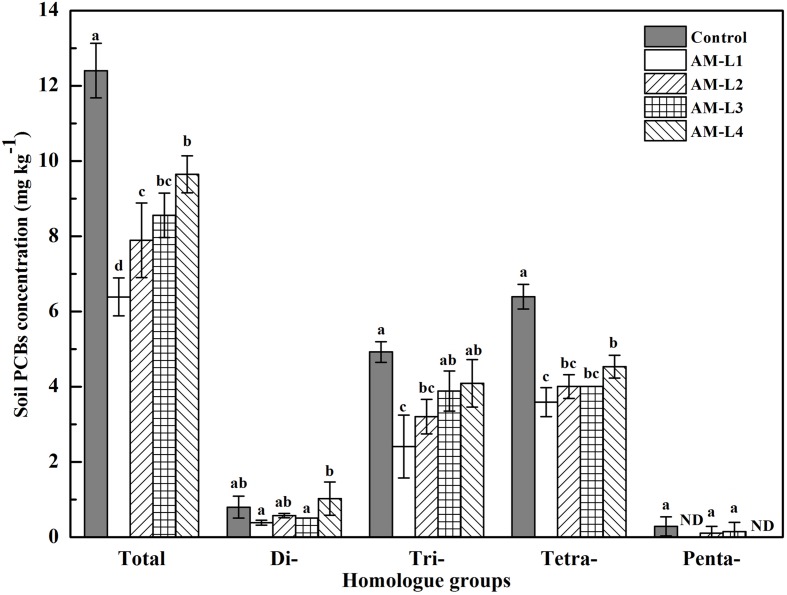
**Concentration of PCB homolog groups in different soil layers of AMF-inoculated treatments and the control after 40 days of *Cucurbita pepo* L. growth**. AM-L1 to AM-L4 were four horizontal soil layers from the central mesh to outer wall in the hyphal compartment of AMF-inoculated treatment, respectively. Bars represent standard errors and different letters above bars indicate significant differences, determined by Duncan’s multiple range test at *P* < 0.05.

After 40 days of incubation, the total PCBs concentration of the rhizobox soil in AMF inoculated treatment was significant lower (*P* < 0.05) than that in the control, indicating the AMF hyphae had beneficial effect on soil PCBs dissipation. Soil Aroclor 1242 dissipation rates ranged from 35.67% of L4 layer to 57.39% of L1 layer in AMF inoculated treatment, in which the L1 layer was significant higher (*P* < 0.05) than other three layers (**Figure 1**). Among the four soil layers, the di-, tri-, and tetrachlorinated biphenyls concentrations were always exhibit the highest value in the L4 layer, and there were significant differences between the L4 and L1 layers (*P* < 0.05) while no statistical differences were found between the L1 and L2 layers. In general, high overall PCB reduction was observed for tri- and tetrachlorinated biphenyls than for di- and pentachlorinated biphenyls. Pearson’s correlation coefficients indicated that the concentrations of soil tri- (*r*^2^ = -0.793), tetrachlorinated biphenyls (*r*^2^ = -0.938) and the total PCBs (*r*^2^ = -0.923) were significantly and negatively correlated with soil hyphal length (*P* < 0.01), while only trichlorinated biphenyl concentration was found negatively correlated with AMF PLFA biomass (*r*^2^ = -0.605, *P* < 0.05). AMF remediation of organic pollutants contaminated soils has been well-studied yet, however, with most of them only focused on the whole mycorrhizosphere effect (Joner and Leyval, 2003; Teng et al., 2010; Lu et al., 2014). To our knowledge, this is the first time to demonstrate the PCBs dissipation in the hyphosphere, and the enhanced dissipation of PCBs could be due to the increased bacterial growth and vitality induced by the external mycelial exudates (Toljander et al., 2007).

**FIGURE 2:**
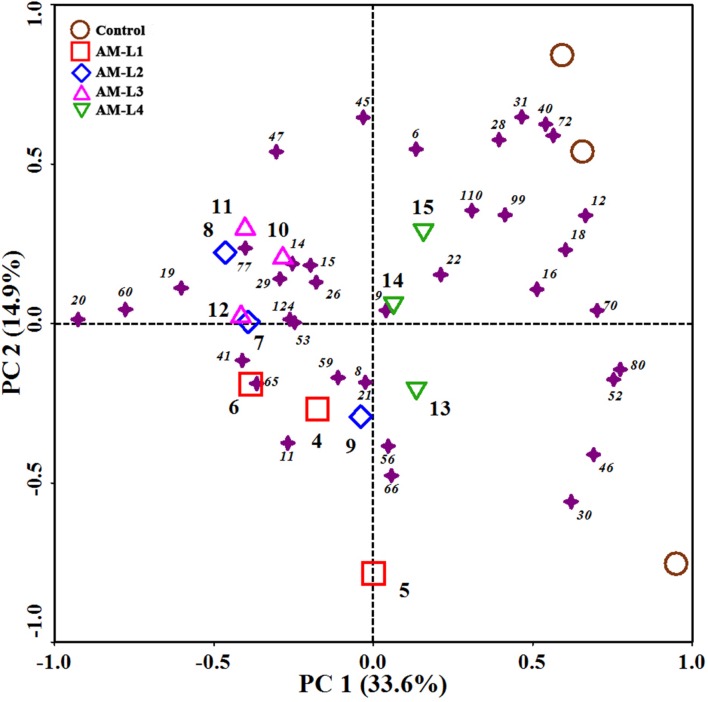
**Principal component analysis (PCA) of PCB congener profiles in different soil layers of AMF-inoculated treatments and the control after 40 days of *Cucurbita pepo* L. growth**. Samples were represented by open symbols while PCB congeners by solid asterisks. Numbers beside asterisks represent IUPAC congeners.

The mycorrhizal hyphae did not only increase PCB dissipation rate, but also alter the soil PCB congener profiles. The PCB congener profiles of different soil layers in the AMF-inoculated treatment were grouped together, and distinctly separated from the control in PC1 (**Figure 2**). Among the different soil layers, the L4 layer was distinguished from the other three layers by positive PC1 values. According to our previous study which indicated that different plant root exudates or secondary metabolites resulted in different PCB congener profiles (Qin et al., 2014b), we can conclude that mycorrhizal hyphal exudates can play an important role in both degrading PCBs and shaping congener profiles.

### PCB-Degrading Related Gene Abundance

The copy numbers of soil bacterial 16S rDNA gene varied from 1.57 ± 0.35 × 10^9^ to 2.44 ± 0.32 × 10^9^g^-1^ dry soil (**Table 2**). The 16S abundance in the L1 and L2 layers of AMF-inoculated treatment were higher, but only the abundance in the L1 layer was significant higher than the other two layers and the control (*P* < 0.05). The hyphosphere could provide important niches for bacterial growth through transferring photo-assimilates into the soil or rapid turnover of mycorrhizal mycelium, in which the exudation of carbohydrates by living hyphae may enable a more direct and reciprocal interaction between mycorrhizal fungi and other microorganisms (Staddon et al., 2003; Toljander et al., 2007).

**Table 2:** Abundances of 16S, *bph*A, *bph*C(*Rh*), and *bph*C(*Ps*) genes in different soil layers under mycorrhiza inoculation treatments and the control (mean ± SD; *n* = 3).

	16S gene (×10^9^ copies g^-1^)	*bphA* gene (×10^4^ copies g^-1^)	*bphC*(*Rh*) (×10^5^ copies g^-1^)	*bphC*(*Ps*) (×10^3^ copies g^-1^)
Control	1.59 ± 0.06 a	6.75 ± 0.65 a	7.15 ± 0.19 a	1.59 ± 0.41 a
AM-L1	2.44 ± 0.32 b	9.43 ± 1.02 b	9.23 ± 0.58 c	2.16 ± 0.98 a
AM-L2	1.89 ± 0.40 ab	7.42 ± 1.15 a	7.84 ± 0.12 ab	1.54 ± 0.50 a
AM-L3	1.57 ± 0.35 a	6.78 ± 0.42 a	8.72 ± 0.74 bc	1.13 ± 0.21 a
AM-L4	1.57 ± 0.33 a	6.59 ± 1.28 a	7.26 ± 0.47 a	1.08 ± 0.08 a

*Different *lowercase letters* in the same column indicate significant differences between the control and different soil layers by Duncan’s multiple range test at *P* < 0.05*.

Several studies have successfully calculated the PCB-degrading bacterial population in various contaminated soils using qPCR technique to target the *bphA* or *bphC* genes (Correa et al., 2010; Petrić et al., 2010; Lu et al., 2014). In the present study, the *bphA* and *bphC* genes were detected by qPCR in both AMF-inoculated treatment and the control. The *bphA* gene population was significant higher in the L1 layer than other soil layers and the control, while no significant difference was observed between the L2-L4 layers and the control (**Table 2**). The population size of *Rhodococcus*-like *bphC* gene [*bphC*(*Rh*)] was found approximately 2–3 orders of magnitude more than that of *Pseudomonas*-like *bphC* gene [*bphC*(*Ps*)], indicating that the *bphC*(*Rh*) gene played a key role in Aroclor 1242 dissipation in this spiked soil. The *bphC*(*Rh*) gene abundance was significant higher in the L1 layer when compared to the control (**Table 2**), and was found correlated significantly with the soil AMF hyphal length (*r*^2^ = 0.624, *P* < 0.05) and PLFA biomass (*r*^2^ = 0.577, *P* < 0.05). Evidences have shown that hyphal exudates from *Glomus* sp. contained low molecular weight (LMW) sugar and organic acids, which could probably be metabolized by bacterial (Toljander et al., 2007). Furthermore, some LMW organic acids, such as salicylate, can act as inducers of PCB degradation by indigenous bacteria (Singer et al., 2003). It can be hypothesized that the mycorrhizal hyphal exudates may contain some LMW organic compounds which could act as inducers of the *bphC*(*Rh*) gene and elevate the gene expression level. This could partly explain the correlation between the *bphC*(*Rh*) gene abundance and the hyphal length. However, it seems that the hyphal exudates of *Glomus mosseae* we used in the present study lack of inducers of *bphC*(*Ps*) gene. As different AMF or host plant may result in different hyphal exudates, further studies are needed to explore potential inducers for *bph* gene expression and elucidate their mechanisms.

### Changes in Microbial Community

A total of 67513 sequences were obtained after passing several specific filters (Yadav et al., 2014; Yang et al., 2014). The relative abundance of the top 14 taxonomic categories at class level is demonstrated in Supplementary Figure S3. The taxonomic classes other than these results are categorized as “Others.” Sequences that do not show homology in the NCBI database have been labeled as “Unclassified.” The *Betaproteobacteria* was largely dominant, representing 12.15–14.26% of the population in all soils. The *Actinobacteria* was the next dominant taxonomy observed in all the soils, followed by the *Alpha*- and *Gammaproteobacteria*. Among the 14 selected classes, the *Anaerolineae* was found negatively correlated with the concentration of dichlorinated biphenyls (*r*^2^ = -0.660, *P* < 0.01) (**Table 3**). Previous studies have revealed that the phylum *Chloroflexi* was one of the several most abundant groups associated with Aroclor 1242 degradation (Mercier et al., 2014) or perchloroethene (PCE) dechloronation (Kittelmann and Friedrich, 2008). Mercier et al. (2014) found that *Chloroflexi* was the second-most abundant phylum shared between three granular activated carbons which were incubated for a month with Aroclor 1242 spiked sediment, and the *Anaerolineae* class was found dominated in this phylum. The strictly anaerobic genera in *Anaerolineae* class could ferment carbohydrates and amino acids around the mycorrhizal hyphae, and may also utilize low chlorinated biphenyls such as dichlorinated biphenyls. Aside from the *Anaerolineae*, the class *Alphaproteobacteria* was also found negatively correlated with the concentration of tetrachlorinated biphenyls (*r*^2^ = -0.525, *P* < 0.05). Most of the *Alphaproteobacteria* were assigned to the *Rhizobiales* and *Rhodospirillales* orders. One possible reason is that certain members of the *Rhizobiales* or *Rhodospirillales* have the ability to biotransform highly chlorinated PCBs (Ahmad et al., 1997; Bertin et al., 2011). Otherwise, there is also the possibility that mycorrhizal hyphal exudates stimulated the growth of *Alphaproteobacteria* and enhanced the co-metabolization of tetrachlorinated biphenyls by PCB degraders consequently.

**Table 3:** Pearson’s correlation coefficients relating PCB congener concentrations and the relative abundance of bacterial groups at the class, order, and family level, respectively.

Classification level	Bacterial group	Pearson’s correlation coefficients
Class	Anaerolineae	Di-CBs (*r*^2^= -0.660^∗∗^)
	Alphaproteobacteria	Tetra-CBs (*r*^2^= -0.525^∗^)
Order	Rhizobiales	Tetra-CBs (*r*^2^= -0.548^∗^)
	Sphingomonadales	Di-CBs (*r*^2^= 0.694^∗∗^)
	Xanthomonadales	Tri-CBs (*r*^2^= 0.566^∗^)
Family	Anaerolineaceae	Di-CBs (*r*^2^= -0.660^∗∗^)
	Comamonadaceae	Tri-CBs (*r*^2^= -0.516^∗^)
	Oxalobacteraceae	Di-CBs (*r*^2^= 0.575^∗^), Tri-CBs (*r*^2^= 0.562^∗^), Penta-CBs (*r*^2^= 0.526^∗^), total PCBs (*r*^2^= 0.623^∗^)
	Streptomycetaceae	Tri-CBs (*r*^2^= 0.545^∗^), Penta-CBs (*r*^2^= 0.521^∗^)

*^∗^*P* < 0.05; ^∗∗^*P* < 0.01*.

At the order level, *Burkholderiales*, *Actinomycetales*, *Sphingobacteriales*, *Bacillales*, *Xanthomonadales*, *Rhizobiales* are dominated, among which the *Burkholderiales* is the most abundant. Offre et al. (2008) reported that subgroups such as *Comamonadaceae*, *Oxalobacteraceae* and *Rubrivivax* affiliated to *Burkholderiales* are preferentially associated with AM. Soil *Burkholderiales* has been shown to be involved in biological suppression of pathogen, plant-growth promotion, and N-fixation (Xu et al., 2015). Our results were in consistent with the previous studies that species of the genus *Burkholderia* were abundant both in rRNA and in rDNA clone libraries generated from the polluted soil (Nogales et al., 2001). The *Burkholderia xenovorans* LB400 has been considered as one model organism to study the aerobic biodegradation of PCBs because of their ability to oxidize wide range of congeners as well as use some congeners as sources of carbon and energy (Tillmann et al., 2005; Parnell et al., 2010). Though no correlation was found between the abundance of *Burkholderiales* and soil PCB congeners concentrations, the *Burkholderiales* was more abundant in the L1 layer of the AMF-inoculated treatment than other layers and the control (*P* < 0.05), indicating mycorrhizal hyphal exudates may benefit the growth of this group of bacteria. The negative correlation between the abundance of *Rhizobiales* and the concentration of tetrachlorinated biphenyls (*r*^2^ = -0.548, *P* < 0.05) were proved (**Table 3**), which provided strong evidence for our above hypothesis about the relationship between *Alphaproteobacteria* and tetrachlorinated biphenyls.

Among the families classified by the RDP classifier, *Chitinophagaceae*, *Xanthomonadaceae*, *Cyanobacteria* Family XIII, *Comamonadaceae* and *Burkholderiaceae* are the most abundant five families. The relative abundance of family *Comamonadaceae* was negatively correlated with trichlorinated biphenyls (*r*^2^ = -0.516, *P* < 0.05) (**Table 3**). The *Comamonadaceae* are a physiologically heterogeneous group of bacteria; they are known to consume a broad spectrum of organic carbon compounds that range from simple sugars to complex aromatic compounds (Kersters et al., 2006). Genera in this family such as *Acidovorax* sp. (formerly *Pseudomonas* sp.) strain KKS102 and *Hydrogenophaga taeniospiralis* IA3-A have the well-known ability of PCB- and biphenyl-degrading or cometabolizing (Lambo and Patel, 2006; Ohtsubo et al., 2006). It is suggested that members of the *Comamonadaceae* may be stimulated by the presence of AMF in PCBs contaminated soil. The results was in accordance with Offre et al. (2007), who found that in the *Medicago truncatula* rhizosphere, the presence of AMF increased the relative abundance of *Comamonadaceae* taxa. However, results from Pivato et al. (2009) and Nuccio et al. (2013) indicated that taxa from *Comamonadaceae* responded negatively to AMF. The mechanisms for these interactions are still unknown, and may result from the direct or indirect manipulation of the community through hyphal exudates (Toljander et al., 2007; Nuccio et al., 2013). Similar to the class *Anaerolineae*, the family *Anaerolineaceae* was also negatively correlated with the concentration of dichlorinated biphenyls (*r*^2^ = -0.660, *P* < 0.01). We also found positive correlations between the relative abundance of family *Oxalobacteraceae* and di- (*r*^2^= 0.575, *P* < 0.05), tri- (*r*^2^= 0.562, *P* < 0.05), pentachlorinated biphenyls (*r*^2^= 0.526, *P* < 0.05) and the total PCB concentrations (*r*^2^= 0.632, *P* < 0.05), while the relative abundance of family *Streptomycetaceae* was correlated with tri- (*r*^2^= 0.545, *P* < 0.05) and pentachlorinated biphenyls (*r*^2^= 0.521, *P* < 0.05) (**Table 3**). Given that these two families were relatively lower in the L1 and L2 layers when compared with the other two layers and the control, we can speculate that the mycorrhizal hyphae may secrete certain inhibitors or compete nutrition with them and resulted in decreased biomass consequently.

**FIGURE 3:**
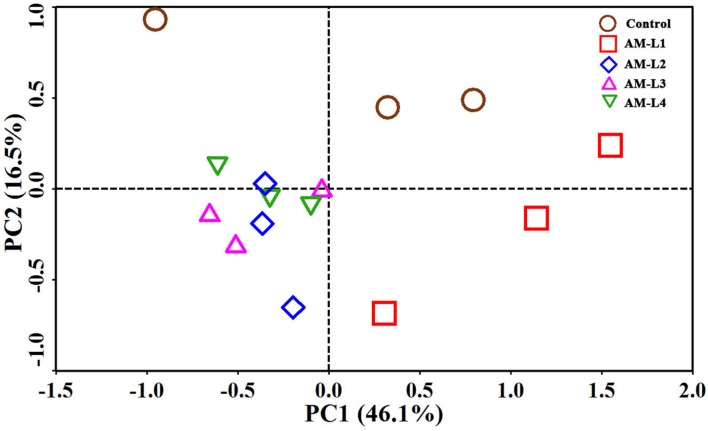
**Principal component analysis of bacterial communities in different soil layers of AMF-inoculated treatments and the control after 40 days of *Cucurbita pepo* L. growth**. AM-L1 to AM-L4 were four horizontal soil layers from the central mesh to outer wall in the hyphal compartment of AMF-inoculated treatment, respectively.

Principal component analysis showed distinct separation of the microbial community from the control in the PC2 to the microbial community that developed under different soil layers of the AMF-inoculated treatment (**Figure 3**). Among the four soil layers in the AMF-inoculated treatment, the L1 layer was distinguished from the other three layers by positive PC1 value, while no obvious difference in microbial community was found between each of the three soil layers. The PCA results strongly supported our hypothesis that the existence as well as the abundance of arbuscular mycorrhizal hyphae could play an important role in shaping the mycorrhizosphere bacterial community which responsible for PCB dissipation.

## Conclusion

This study demonstrates the important role of arbuscular mycorrhizal hyphae in degradative efficiency of different PCB congeners. Analysis of bacterial growth, *bph* gene abundance, and bacterial community composition were also shown to vary with different soil mycorrhizal hyphal biomass. The results strongly supported our previous hypothesis that the mycorrhizal hyphae could accelerate the dissipation of low-chlorinated biphenyls as well as shaping the soil PCB congener profiles via altering bacterial growth and community compositions in the mycorrhizosphere. As the AMF-plant symbiosis is ubiquitous in terrestrial ecosystems, the influence of AMF on PCB congener dissipation is broadly relevant across terrestrial ecosystems for the remediation of PCB contaminated soils. These data improved our understanding of mycorrhizal hyphae-bacteria interactions in PCB dissipation. Further investigation is needed to identify the PCBs degraders in AM fungal hyphosphere soil, and understand the metabolic pathways involved in PCBs degradation.

## Author Contributions

HQ: conceived and designed the work that led to the submission, acquired data, and played an important role in interpreting the results; PB: revised the manuscript; JX: revised the manuscript and approved the final version.

## Conflict of Interest Statement

The authors declare that the research was conducted in the absence of any commercial or financial relationships that could be construed as a potential conflict of interest.

## Footnotes

1 http://www.qiime.org

2 http://www.arb-silva.de/download/archive/qiime/
