# Supplementary material for: Arbuscular Mycorrhizal Fungal Hyphae Alter Soil Bacterial Community and Enhance Polychlorinated Biphenyls Dissipation
Source: Front Microbiol. 2016 Jun 15;7:939. doi: 10.3389/fmicb.2016.00939 (PMC4908113; doi:10.3389/fmicb.2016.00939)

**Figure S1** Schematic view of *Cucurbita pepo* L. roots and mycorrhizal hyphae in the two-compartment rhizobox.

**Figure S2** Reprsentative GC-MS chromatograph for PCB congeners profile in non-AM control soil.

**Figure S3** Classification of bacterial diversity at Class, Order and Family level of different soil layers of AMF-inoculated treatments and the non-AM control in terms of relative abundance. The abundance has been represented in terms of percentage in total effective bacterial sequences in a soil.

**Figure S1**

**
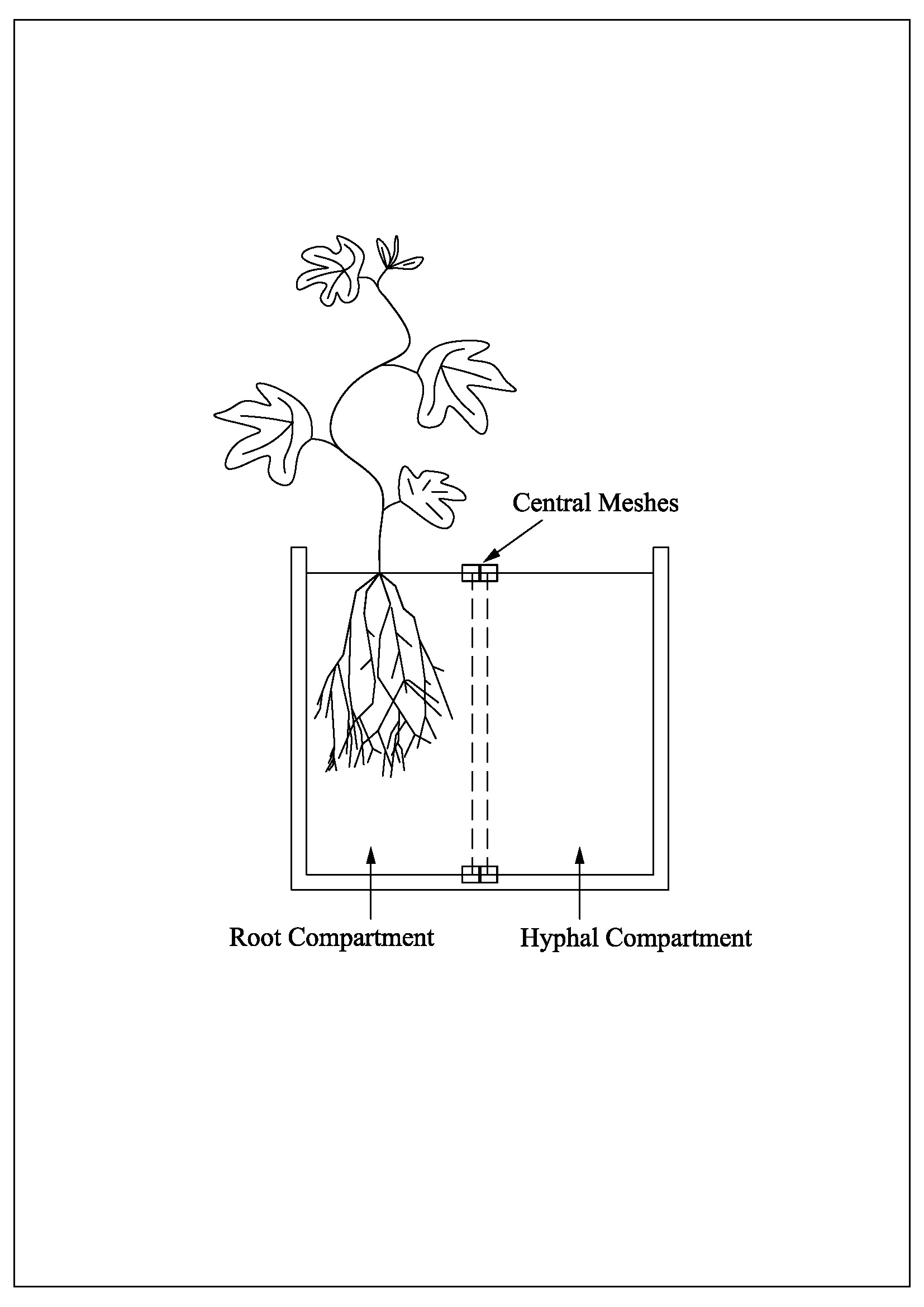
**

**Figure S2**


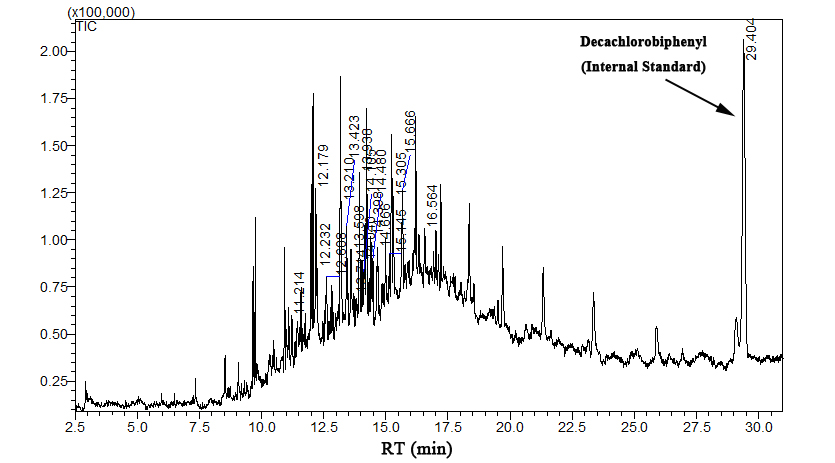


| **Retention time (min)** | **IUPAC No.** | **PCB congener** |
| --- | --- | --- |
| 11.214 | 8 | 2,4'-dichloro-1,1'-Biphenyl |
| 12.179 | 16 | 2,2',3-trichloro-1,1'-Biphenyl |
| 12.232 | 11 | 3,3'-dichloro-1,1'-Biphenyl |
| 12.609 | 18 | 2,2',5-trichloro-1,1'-Biphenyl |
| 13.21 | 31 | 2,4',5-trichloro-1,1'-Biphenyl |
| 13.423 | 31 | 2,4',5-trichloro-1,1'-Biphenyl |
| 13.598 | 30 | 2,4,6-trichloro-1,1'-Biphenyl |
| 13.714 | 46 | 2,2',3,6'-tetrachloro-1,1'-Biphenyl |
| 13.938 | 45 | 2,2',3,6-Tetrachloro-1,1'-Biphenyl |
| 14.04 | 52 | 2,2',5,5'-tetrachloro-1,1'-Biphenyl |
| 14.105 | 80 | 3,3',5,5'-tetrachloro-1,1'-Biphenyl |
| 14.398 | 40 | 2,2',3,3'-tetrachloro-1,1'-Biphenyl |
| 14.48 | 18 | 2,2',5-trichloro-1,1'-Biphenyl |
| 14.666 | 52 | 2,2',5,5'-tetrachloro-1,1'-Biphenyl |
| 15.145 | 70 | 2,3',4',5-tetrachloro-1,1'-Biphenyl |
| 15.305 | 70 | 2,3',4',5-tetrachloro-1,1'-Biphenyl |
| 15.667 | 72 | 2,3',5,5'-tetrachloro-1,1'-Biphenyl |
| 16.564 | 99 | 2,2',4,4',5-pentachloro-1,1'-Biphenyl |
| 29.404 | 209 | 2,2´,3,3´,4,4´,5,5´,6,6´-Decachloro-1,1'-Biphenyl |

**Figure S3**


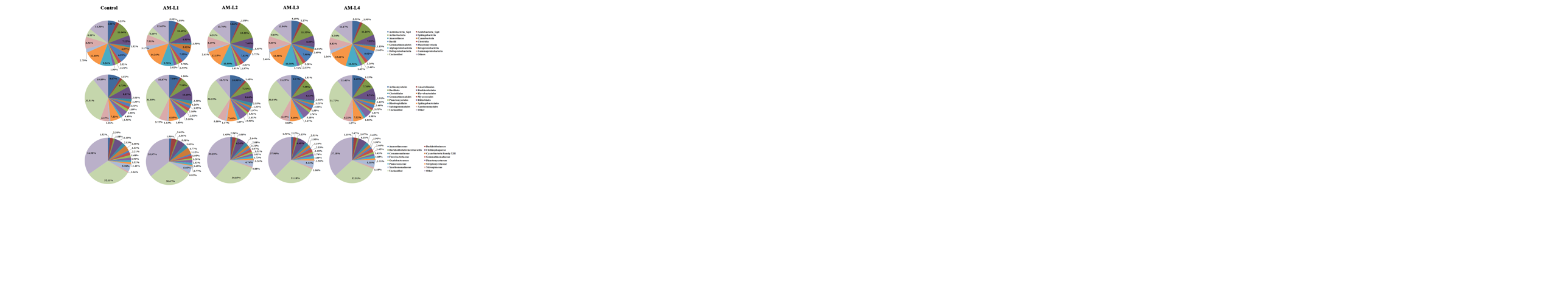

Supplement: Supplementary file 1 [file Data_Sheet_1.DOC]
